# Deficiency and haploinsufficiency of histone macroH2A1.1 in mice recapitulate hematopoietic defects of human myelodysplastic syndrome

**DOI:** 10.1186/s13148-019-0724-z

**Published:** 2019-08-22

**Authors:** Oxana Bereshchenko, Oriana Lo Re, Fedor Nikulenkov, Sara Flamini, Jana Kotaskova, Tommaso Mazza, Marguerite-Marie Le Pannérer, Marcus Buschbeck, Cesarina Giallongo, Giuseppe Palumbo, Giovanni Li Volti, Valerio Pazienza, Libor Cervinek, Carlo Riccardi, Lumir Krejci, Sarka Pospisilova, A. Francis Stewart, Manlio Vinciguerra

**Affiliations:** 1 Department of Medicine, Department of Philosophy, Social Sciences and Education, University of Perugia, Perugia, Italy; 2 International Clinical Research Center, St’Anne University Hospital, Brno, Czech Republic; 3 Department of Biology, Faculty of Medicine, Masaryk University, Brno, Czech Republic; 4 Central European Institute of Technology, Masaryk University, Brno, Czech Republic; 5 Department of Internal Medicine - Hematology and Oncology, Faculty of Medicine, University Hospital Brno and Masaryk University, Brno, Czech Republic; 6 IRCCS Casa Sollievo della Sofferenza, Bioinformatics unit, San Giovanni Rotondo, Italy; 7 Josep Carreras Leukemia Research Institute (IJC), Universitat Autònoma de Barcelona, Campus ICO-Germans Trias I Pujol, Badalona, Spain; 8 Programme of Predictive and Personalized Medicine of Cancer, Germans Trias i Pujol Research Institute (PMPPC-IGTP), Badalona, Spain; 9 Division of Hematology, A.O.U. Policlinico-OVE, University of Catania, Catania, Italy; 10 Department of Medical and Surgical Sciences and Advanced Technologies “GF Ingrassia”, University of Catania, Catania, Italy; 11 Department of Biomedical and Biotechnological Sciences, University of Catania, Catania, Italy; 12 Gastroenterology unit, IRCCS Casa Sollievo della Sofferenza, San Giovanni Rotondo, Italy; 13 Genomics, Biotechnology Center, Center for Molecular and Cellular Bioengineering, Technische Universität Dresden, Dresden, Germany

**Keywords:** Hematopoiesis, MacroH2A1, Myelodysplastic syndrome

## Abstract

**Background:**

Epigenetic regulation is important in hematopoiesis, but the involvement of histone variants is poorly understood. Myelodysplastic syndromes (MDS) are heterogeneous clonal hematopoietic stem cell (HSC) disorders characterized by ineffective hematopoiesis. MacroH2A1.1 is a histone H2A variant that negatively correlates with the self-renewal capacity of embryonic, adult, and cancer stem cells. MacroH2A1.1 is a target of the frequent U2AF1 S34F mutation in MDS. The role of macroH2A1.1 in hematopoiesis is unclear.

**Results:**

MacroH2A1.1 mRNA levels are significantly decreased in patients with low-risk MDS presenting with chromosomal 5q deletion and myeloid cytopenias and tend to be decreased in MDS patients carrying the U2AF1 S34F mutation. Using an innovative mouse allele lacking the macroH2A1.1 alternatively spliced exon, we investigated whether macroH2A1.1 regulates HSC homeostasis and differentiation. The lack of macroH2A1.1 decreased while macroH2A1.1 haploinsufficiency increased HSC frequency upon irradiation. Moreover, bone marrow transplantation experiments showed that both deficiency and haploinsufficiency of macroH2A1.1 resulted in enhanced HSC differentiation along the myeloid lineage. Finally, RNA-sequencing analysis implicated macroH2A1.1-mediated regulation of ribosomal gene expression in HSC homeostasis.

**Conclusions:**

Together, our findings suggest a new epigenetic process contributing to hematopoiesis regulation. By combining clinical data with a discrete mutant mouse model and in vitro studies of human and mouse cells, we identify macroH2A1.1 as a key player in the cellular and molecular features of MDS. These data justify the exploration of macroH2A1.1 and associated proteins as therapeutic targets in hematological malignancies.

**Electronic supplementary material:**

The online version of this article (10.1186/s13148-019-0724-z) contains supplementary material, which is available to authorized users.

## Background

The healthy hematopoietic stem cell (HSC) pool is maintained by genetically and epigenetically regulated gene expression [1, 2]. When these systems go awry, aberrant hematopoietic stem and progenitor cells may emerge (reviewed in [3]), which are implicated in the development of a heterogeneous group of disorders called myelodysplastic syndromes (MDS). MDS are characterized by ineffective hematopoiesis, leading to variable peripheral blood cytopenias and risk of transformation to acute myeloid leukemia (AML) [4]. MDS can remain stable for many years with few symptoms or can rapidly progress into a more aggressive MDS subtype [5]; current treatment options, however, are limited and often ineffective. Studies have started to identify common mutations underlying HSC dysregulation in MDS, but more progress is needed to identify the molecules and pathways contributing to disease mechanisms.

Recent data have suggested a possible role for histone variants, in particular, the macroH2A1 splice variant macroH2A1.1, in the pathological processes underlying MDS development [6]. Histone macroH2A1 exists as two mutually exclusive exon-spliced isoforms: macroH2A1.1 and macroH2A1.2. These isoforms display largely divergent functions in cell differentiation and tumorigenesis [7, 8]. MacroH2A1.1 is a potent transcriptional modulator associated with favorable outcomes in several solid tumor types, restriction of cancer stem-cell emergence, and PARP1 (Poly (ADP-ribose) Polymerase-1)-dependent chromatin remodeling [8–20]. The gene encoding human macroH2A1.1 and macroH2A1.2, *H2AFY*, is found on the long arm of chromosome 5 (at 5q31.1), which is deleted in ~ 20% of MDS patients. Between 45 and 85% of MDS patients exhibit mutations in various components of the RNA spliceosome machinery, including *U2AF1*, *ZRSR2*, *SRSF2*, and *SF3B1* [21]. Recapitulation of the common *U2AF1 S34F* mutation in mice reproduces aspects of human MDS and induces dysregulated splicing of multiple gene products, including *H2AFY* [22]. Yip et al. recently demonstrated that bone marrow (BM) progenitor cells from MDS patients with an endogenous *U2AF1 S34F* mutation showed aberrantly spliced *H2AFY*, resulting in reduced macroH2A1.1, but not macroH2A1.2, isoform expression in both erythroid and granulomonocytic derived colonies [6]. shRNA-mediated silencing of macroH2A1.1 in BM progenitor cells led to increased apoptosis and decreased differentiation when cells were cultured under erythroid and granulomonocytic conditions [6].

Here, we report for the first time that loss of macroH2A1.1 expression in mice led to the dysregulation of myeloid differentiation. RNA-sequencing identified defective production of ribosomal mRNAs as a potential mechanism for disrupted HSC homeostasis following macroH2A1.1 depletion. Thus, macroH2A1.1 delineates its function in HSC homeostasis and differentiation in mice. Moreover, we found that macroH2A1 isoforms’ mRNA levels are significantly decreased in MDS patients with a 5q deletion compared to other MDS groups and healthy individuals.

## Results

### Mice lacking macroH2A1.1 exhibit blood abnormalities under a steady state

**Fig. 1:**
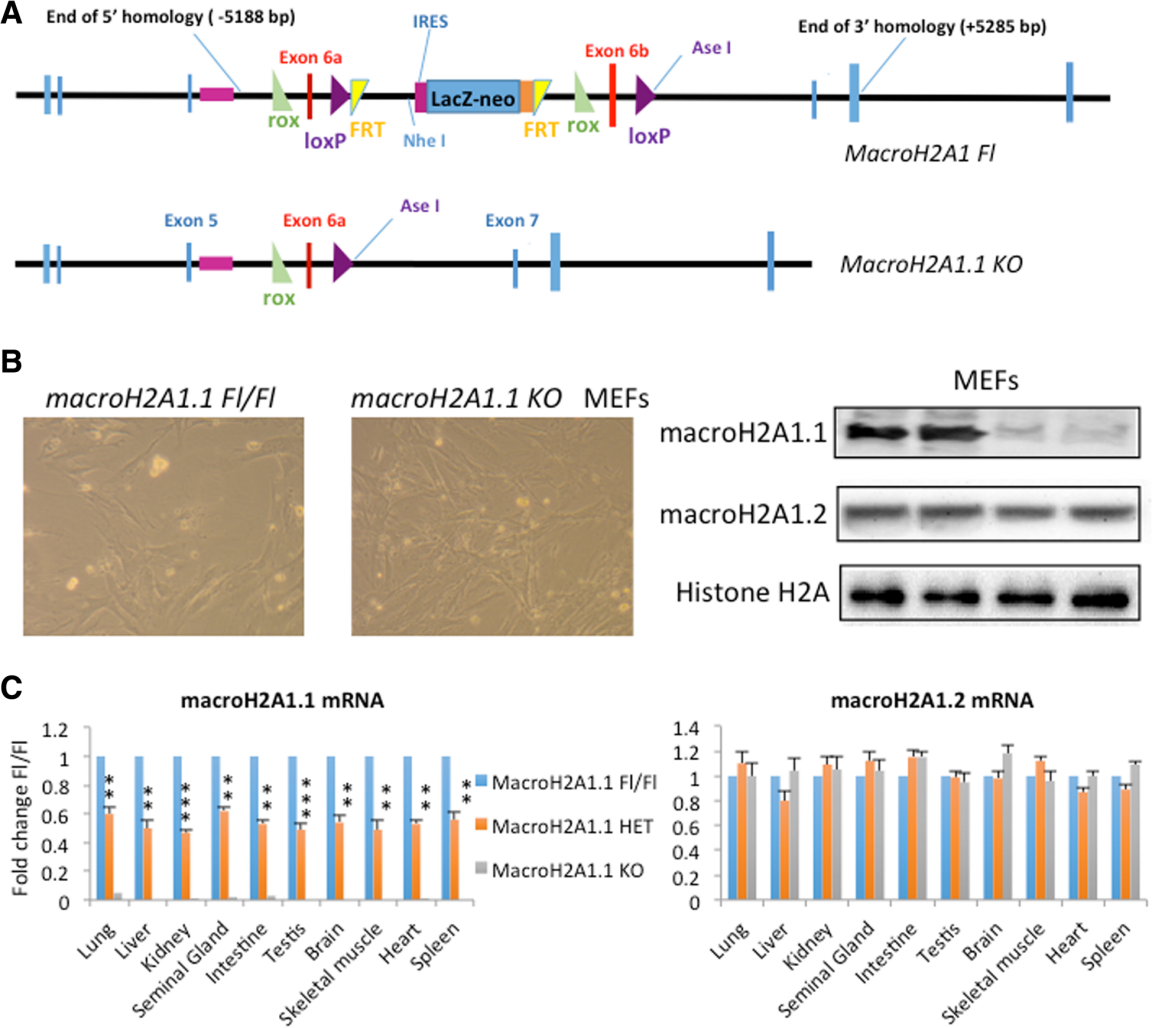
Conditional macroH2A1.1 knockout (KO) in mice. **a** Upper panel. Targeting construct containing the sequence encoding mouse macroH2A1 (H2AFY), a loxP-flanked neomycin (neo) cassette 3′ of exon 6b (included in macroH2A1.2), and a rox-flanked cassette 3′ of exon 6a (included in macroH2A1.1). Lower panel. Targeting construct upon Cre-mediated excision. **b** Left: phase-contrast of first passage mouse embryonic fibroblasts (MEFs) after 3 days in culture. Right: protein lysates from MEFs of macroH2A1.1^fl/fl^ and macroH2A1.1^−/−^ (KO) mice were immunoblotted with anti-macroH2A1.1 and macroH2A1.2 antibodies. Histone H2A was used as a loading control. **c** macroH2A1.1 and macroH2A1.2 mRNA levels in adult mice tissues. RNA was extracted from the lung, liver, kidney, seminal gland, intestine, testis, brain, skeletal muscle, heart, and spleen of 3-month-old macroH2A1.1^fl/fl^, macroH2A1.1^fl/−^ (HET), and macroH2A1.1^−/−^ (KO) mice, and analyzed by qRT-PCR using isoform-specific primers. *N* = 5 mice/group. **p < 0.01; ***p < 0.001 relative to macroH2A1.1^fl/fl^

As macroH2A1.1 expression is decreased in *U2AF1 S34F* MDS patients and its knockdown in vitro perturbs erythroid and granulomonocytic differentiation [6], we sought to investigate macroH2A1.1 function in mice using a gene KO approach. We first generated a mouse line carrying a conditional macroH2A1 allele that permits selective elimination of one of the two macroH2A protein isoforms using either Cre or Dre recombinase (Fig. 1a).

Mice carrying the targeted (floxed; fl) allele exhibited no phenotype compared to wild-type (WT) mice and were viable and fertile (*data not shown*). When crossed to deleter HPRT-Cre mice (129S1/Sv-Hprt^tm1(CAG-cre)Mnn^/J) that ubiquitously express Cre in early development [23], the loxP-flanked cassette and exon 6b were removed thereby generating macroH2A1.1^fl/−^ mice. These mice were then intercrossed to generate macroH2A1.1^−/−^ (KO) mice. MacroH2A1.1^fl/−^ and macroH2A1.1^−/−^ mice were morphologically and histologically indistinguishable from their WT and macroH2A1.1^fl/fl^ littermates under normal conditions, with no overt metabolic, reproductive, or behavioral abnormalities. To confirm the absence of macroH2A1.1 in the KO cells, we isolated murine embryonic fibroblasts (MEFs) from macroH2A1.1^fl/fl^ and macroH2A1.1^−/−^ mice, which exhibited comparable cell morphology (Fig. 1b, left panel), and analyzed extracted protein lysates by Western blotting. MacroH2A1.1, but not macroH2A1.2, protein expression was abolished in macroH2A1.1^−/−^ KO MEFs (Fig. 1b). We then confirmed undetectable macroH2A1.1 mRNA levels in a panel of tissues isolated from adult (3-month-old) macroH2A1.1^−/−^ mice, compared to macroH2A1.1^fl/fl^ littermates, and reduced macroH2A1.1 mRNA levels by 50% in macroH2A1.1^fl/−^ heterozygous (HET) mice (Fig. 1c). MacroH2A1.2 transcript abundance was comparable between all macroH2A1.1 genotypes (Fig. 1c). These results confirmed efficient Cre-mediated macroH2A1.1 allele targeting.

Blood counts and biochemical tests identified a low hematocrit and reduced neutrophil counts in macroH2A1.1^fl/−^ HET and macroH2A1.1^−/−^ KO mice compared to macroH2A1.1^fl/fl^ and WT littermates; an opposite trend was observed for lymphocyte and eosinophil cell counts (Additional file 1: Figure S2A and B). No difference in cholesterol or triglyceride levels was observed between groups, but decreased liver transaminase levels were observed in macroH2A1.1^fl/−^ HET and macroH2A1.1^−/−^ KO mice (Additional file 1: Figure S2C). The blood count abnormalities are reminiscent of those observed in human patients with MDS. We also detected lower alanine transaminase (ALT) and aspartate transaminase (AST) levels in macroH2A1.1^fl/−^ HET, and to a greater extent in macroH2A1.1^−/−^ KO mice (Additional file 1: Figure S2C), which in humans is associated with higher all-cause mortality [24, 25].

### MacroH2A1.1 haploinsufficiency or deficiency perturbs hematopoiesis following DNA damage

**Fig. 2:**
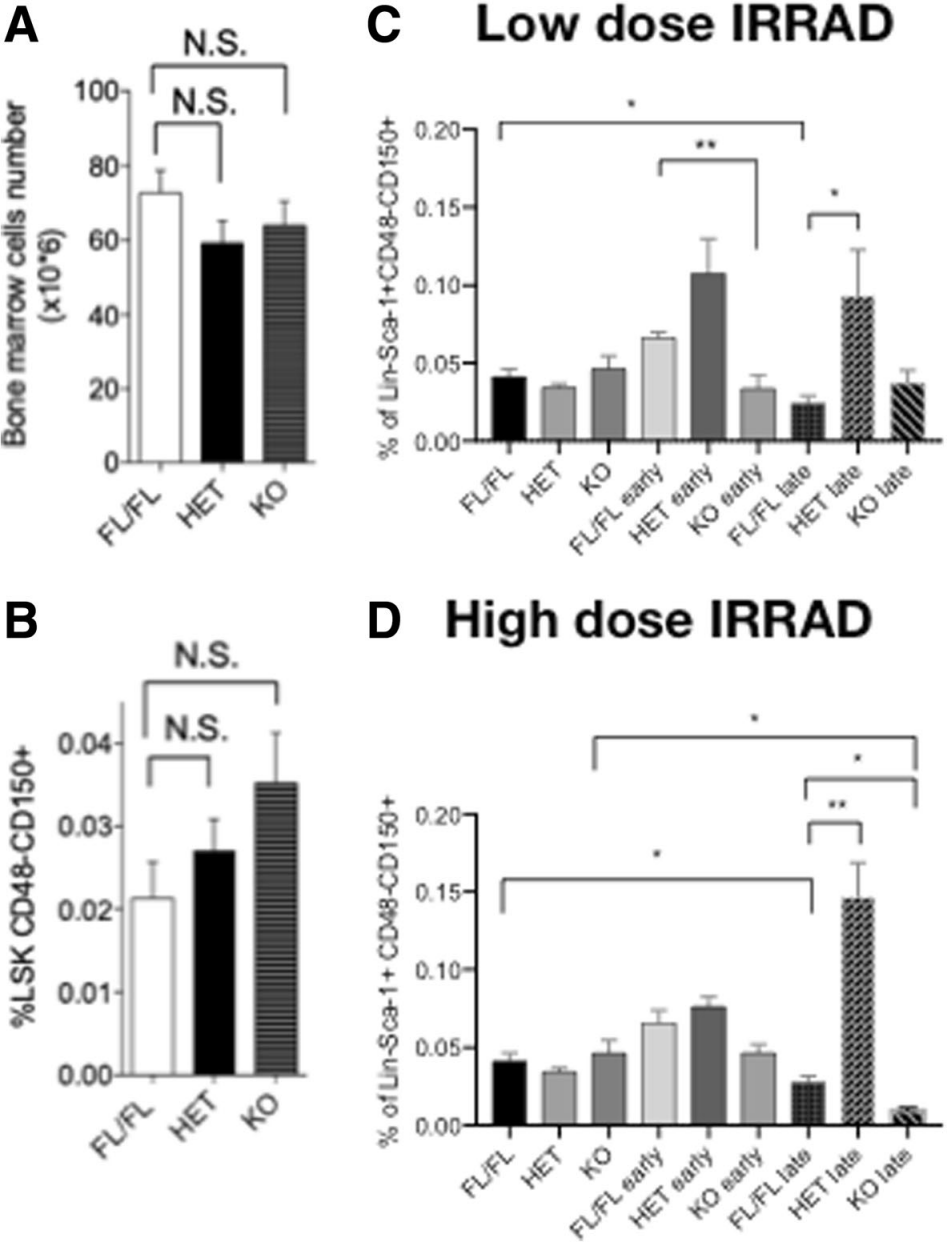
Assessment of the long-term hematopoietic stem-cell (LT-HSC) pool at a steady state and upon irradiation in macroH2A1.1^fl/−^ (HET) and macroH2A1.1^−/−^ (KO) mice. Live, Lin^−^ cells were gated and analyzed for c-Kit and Sca-1 surface marker expression. Lin^−^c-Kit^+^Sca-1^+^ (LSK) cells were gated and further analyzed for CD150^+^ CD48^−^ surface marker expression. Evaluation of the absolute cell number (**a**) and frequencies (percentage relative to whole bone marrow cell count) (**b**) of LT-HSCs in the bone marrow (BM) of macroH2A1.1^fl/fl^, macroH2A1.1^fl/−^ (HET), and macroH2A1.1^−/−^ (KO) mice, at a steady state. *N* = 5–6 mice per genotype. **c**, **d** Evaluation of the absolute cell number and frequencies of LT-HSCs in the BM of irradiated [(600 rad (low dose, **c**) or 1200 rad (high dose, **d**)] macroH2A1.1^fl/fl^, macroH2A1.1^fl/−^ (HET), and macroH2A1.1^−/−^ (KO) mice. Analyses were performed on days 1 (early time point) or 7 (late time point) after irradiation. Error bars represent the SD. *N* = 3–4 mice per genotype. *p < 0.05, **p < 0.01 relative to macroH2A1.1^fl/fl^

An expansion of phenotypically primitive HSCs is often observed in patients with MDS [3]. Murine HSCs are enriched in the bone marrow cell population that do not express mature hematopoietic cell lineage markers (Lin^−^), such as B220, CD4, CD8, Gr-1, Mac-1, and Ter-119, but do express c-Kit and Sca-1, collectively termed LSK (Lin^−^Sca-1^+^c-Kit^+^) cells [26]. HSC and early progenitors can be further characterized within the LSK population based on the surface expression of SLAM (signaling lymphocyte activation molecule) markers CD150 and CD48 [27]. Given the enhanced susceptibility of macroH2A1.1 KO mice to radiation-induced death and cell damage, we tested the effects of haploinsufficiency and absence of the macroH2A1.1 on the hematopoietic compartment at a steady state and under DNA damaging conditions. At a steady state, total bone marrow cellularity (Fig. 2a) as well as the frequency of the LSK CD48^−^CD150^+^ HSC did not vary significantly among macroH2A1.1^fl/fl^, HET and KO mice (Fig. 2b). Because macroH2A1 isoforms regulate self-renewal and differentiation of induced pluripotent stem cells, embryonic stem cells and cancer stem cells [11, 16, 28], we hypothesized that macroH2A1.1 might be involved in regulating the hematopoietic compartment following genotoxic insult in a dose-dependent and time-dependent manner. We analyzed HSCs in the BM of all three macroH2A1.1 genotypes at 1 day (early time point) and 7 days (late time point) after systemic exposure to 600 (low dose) or 1200 rad (high dose) irradiation. Downregulation of c-Kit cell surface expression on functional HSCs has been reported in the situation of distress, such as 5-FU treatment and Myc gene deletion [29]. Since all Lin^−^Sca-1^+^CD150^+^ cells in control animals are c-Kit^+^, the Lin^−^Sca-1^+^CD150^+^CD48^−^ marker combination can also be used to identify HSCs [29]. Since in irradiated mice, we also observed a dramatic reduction in c-Kit^+^ cells (*data not shown*), we used the Lin^−^Sca-1^+^CD150^+^CD48^−^ marker combination to identify HSC in WT, HET, and KO mice following different doses of irradiation in comparison to the same population in untreated mice. We observed that the macroH2A1.1 haploinsufficiency caused a significant increase in HSC frequency at late time point following low-dose (Fig. 2c) and high-dose (Fig. 2d) irradiation compared to WT mice. Conversely, the macroH2A1.1^−/−^ mice showed a decrease in HSC frequency, caused by low (Fig. 2c, early time point) and high (Fig. 2d) doses of irradiation, compared to untreated and similarly treated WT mice. Altogether, these data demonstrate that the macroH2A1.1 isoform regulates HSC response to irradiation in a time- and dose-dependent manner.

### MacroH2A1.1 haploinsufficiency or deficiency in HSCs leads to a myeloid differentiation bias in transplantation assays

**Fig. 3:**
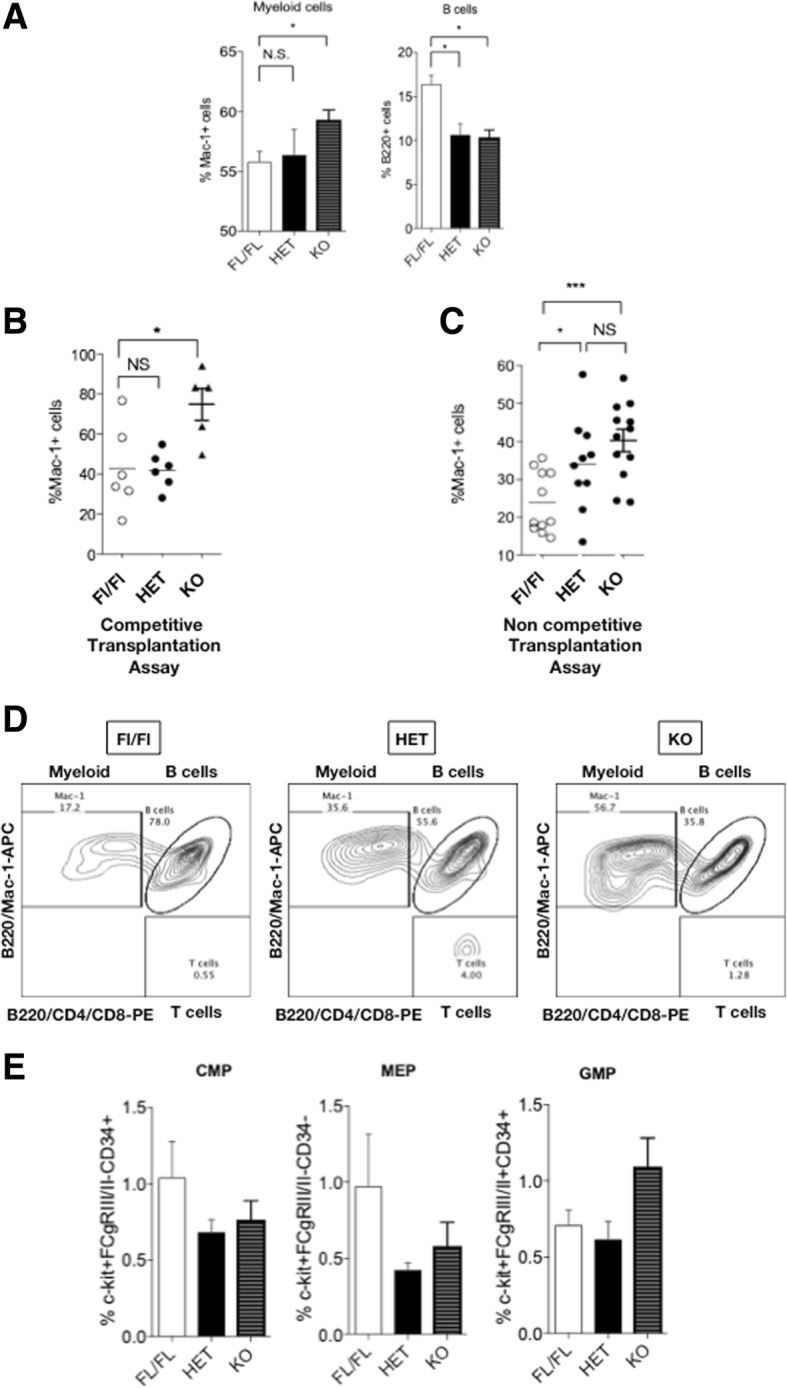
MacroH2A1.1 deficiency causes myeloid bias upon bone marrow (BM) transplantation. **a** Peripheral blood (PB) analysis from macroH2A1.1^fl/fl^, macroH2A1.1^fl/−^ (HET), and macroH2A1.1^−/−^ (KO) mice showing the percentage of myeloid-cell (Mac-1^+^) and B-cell (B220^+^) gated populations. **b**, **c** Dot plots of PB analysis from animals transplanted with macroH2A1.1^fl/fl^, macroH2A1.1^fl/−^ (HET), and macroH2A1.1^−/−^ (KO) BM cells in a competitive (**b**) or noncompetitive (**c**) transplantation assay. Both plots show the percentage of myeloid cells (Mac-1^+^) within engrafted CD45.2^+^ cells. **d** Representative plots indicating in each quadrant the frequency of gated cell populations within CD45.2^+^ cells. Graphs show the frequency of [Mac-1^+^, B (B220^+^), and T (CD4^+^/CD8^+^) cells] within CD45.2^+^ cells. **e** Frequencies of CMPs, MEPs, and GMPs in the BM of irradiated macroH2A1.1^fl/fl^, macroH2A1.1^fl/−^ (HET), and macroH2A1.1^−/−^ (KO) mice. Graphs represent CMP (Lin^−^c-Kit^+^Sca-1^−^CD34^+^CD16/32), MEP (Lin^−^c-Kit^+^Sca-1^−^CD34^−^CD16/32^−^), and GMP (Lin^−^c-Kit^+^Sca-1^−^CD34^+^CD16/32^+^) frequencies in the BM. Results are presented as the means ± SEM of three independent experiments. *N* = 6–10/group. *p < 0.05, ***p < 0.001, NS = not significant

A myeloid differentiation bias is found in human patients with MDS [3]. To understand the downstream effects of macroH2A1.1 haploinsufficiency or deficiency on HSC/hematopoietic progenitor cells (HPC) populations, we analyzed the BM from macroH2A1.1^fl/fl^, macroH2A1.1^fl/−^ HET, and macroH2A1.1^−/−^ KO mice by flow cytometry. Higher frequencies of myeloid (Mac-1^+^) cells were found in macroH2A1.1^−/−^ KO mice compared to macroH2A1.1^fl/fl^ mice, and lower frequencies of B (B220^+^) cells were found in both macroH2A1.1^fl/−^ HET and in macroH2A1.1^fl/fl^ mice (Fig. 3a). To demonstrate that the cause of this myeloid bias observed in macroH2A1.1^−/−^ KO mice is intrinsic to the hematopoietic system, we performed an in vivo transplantation study. Here, flow cytometry analysis confirmed higher frequencies of myeloid cells and lower frequencies of B cells in the peripheral blood of mice receiving macroH2A1.1^fl/−^ HET or macroH2A1.1^−/−^ KO BM cells, compared to mice receiving macroH2A1.1^fl/fl^ BM cells (Fig. 3b–d). The myeloid differentiation bias was also observed in HET mice in noncompetitive BM transplantation assay (Fig. 3c). Moreover, the analysis of the frequencies of myeloid progenitor sub-populations revealed a tendency increase in GMPs (Lin/IL7R/Sca-1^−^c-Kit^+^CD34^+^CD16/32^+^) and a tendency decrease in megakaryocyte-erythrocyte progenitors (MEPs; Lin^−^/IL7R/Sca-1^−^c-Kit^+^CD34^−^CD16/32^−^) in the BM of macroH2A1.1^fl/−^ HET and macroH2A1.1^−/−^ KO mice compared to macroH2A1.1^fl/fl^ mice (Fig. 3e). These data show that decreased macroH2A1.1 levels has a profound impact on HSC differentiation in the BM, resulting in a myeloid skewing similar to that observed in human MDS.

### Downregulation of ribosomal protein genes after macroH2A1.1 depletion

**Fig. 4:**
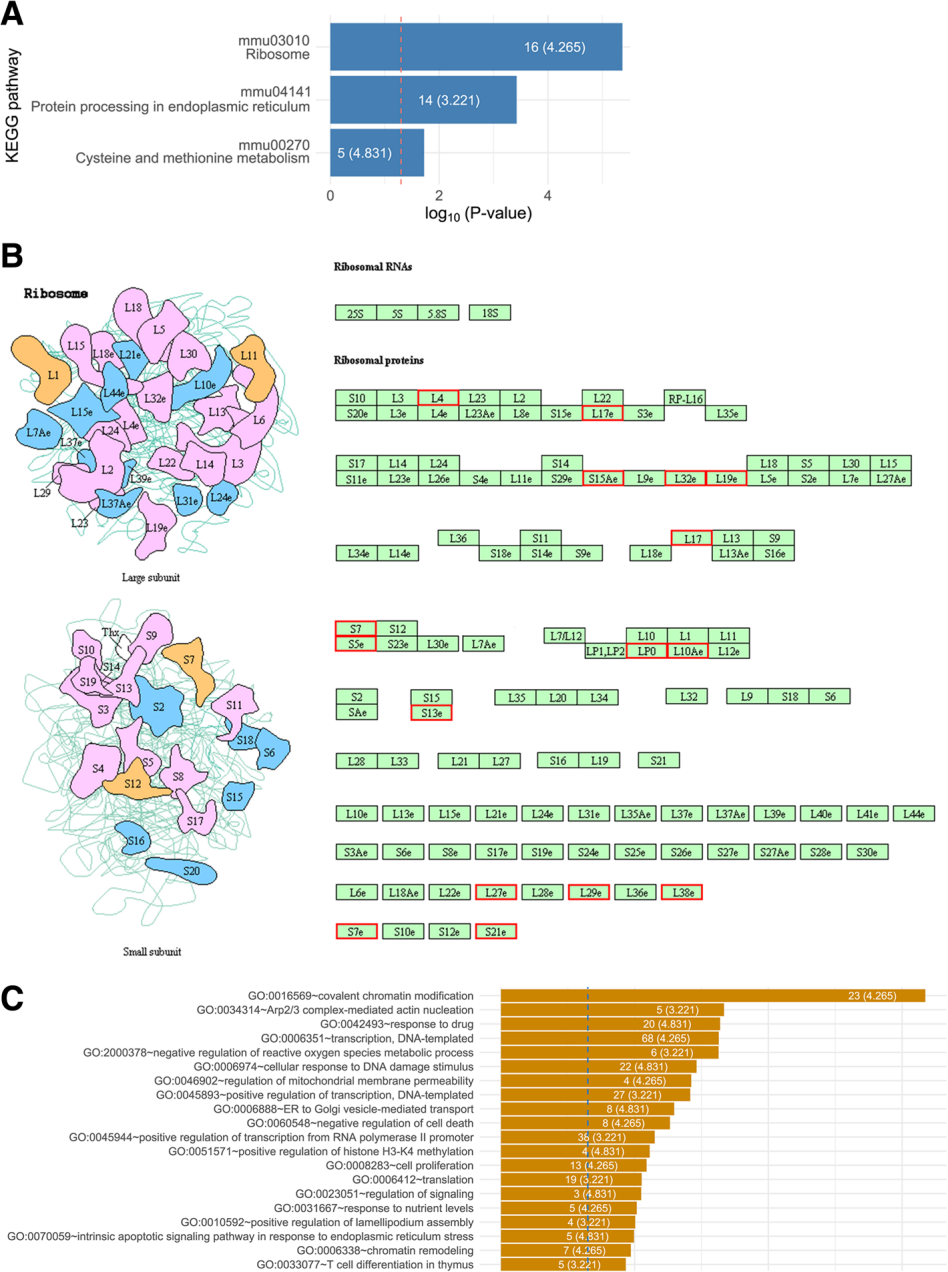
RNA-Seq and bio-informatic analyses on multipotent progenitor cells (CD150^−^CD48^+^ LSK) isolated from bone marrow and using flow cytometry. **a** KEGG pathways significantly represented by the differentially expressed genes. Enrichment-log (*p* values) is reported on the *x*-axis. The dashed red line marks the significant enrichment threshold (*p* value = 0.05). **b** Mouse KEGG map (mmu03010) of the Ribosome pathway. **c** Top 20 most enriched Gene Ontology biological processes. The blue line marks the significance enrichment threshold (*p* value = 0.05), and text within a bar refers to the number of significantly expressed genes belonging to a process and the overall enrichment score (into brackets)

**Fig. 5:**
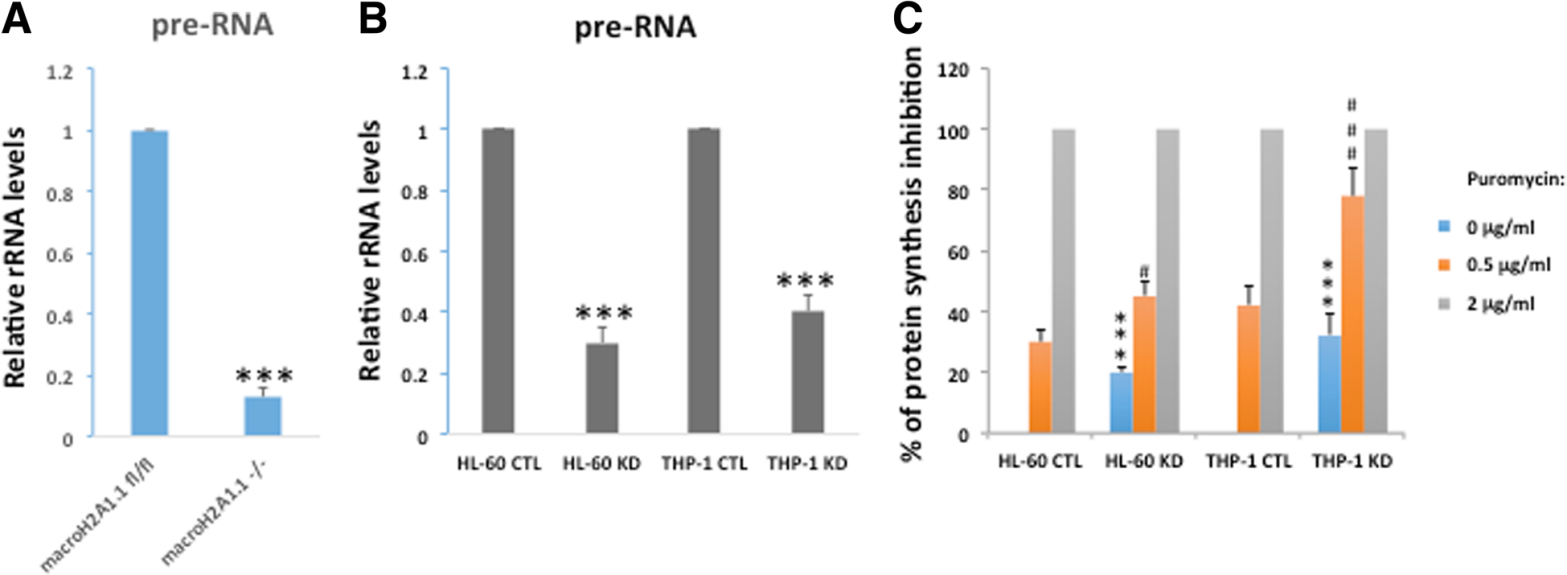
MacroH2A1.1 deficiency decreases rRNA transcription and protein translation in mouse HPCs (**a**) and in human HL-60 and THP-1 cells (**b**), respectively. **a**, **b** Total RNA was extracted and 47S pre-rRNA levels were measured by qPCR in macroH2A1.1^fl/fl^ versus macroH2A1.1^−/−^ HPCs and in HL-60 or THP-1 control (CTL) cells compared to KD cells for macroH2A1.1, respectively. Data were normalized to the amount of GAPDH mRNA and to the amount of 47S pre-rRNA in macroH2A1.1^fl/fl^ or in CTL cells, respectively. **c** Global protein synthesis in HL-60 and THP-1 cells treated for 72 h with puromycin (0, 0.5, 2 μγ/ml) was measured by [3H]-leucine incorporation assay and shown as a percentage of inhibition of control in CTL and macroH2A1.1 KD HL-60 and THP-1 cells. *n* = 3. ***p < 0.001 relative to the respective CTL (0 μg/ml puromycin); ^#^*p* < 0.05, ^###^*p* < 0.001 relative to the respective 0.5 μg/ml puromycin condition

To gain a mechanistic insight into the hematopoietic derangements associated with decreased macroH2A1.1 expression, we performed transcriptome-wide RNA sequencing (RNA-Seq) to identify differentially expressed genes (DEGs) between HPCs (CD150^−^CD48^+^ LSK) isolated by cell sorting from macroH2A1.1^−/−^ KO and macroH2A1.1^fl/fl^ mice. Using a 1.5-fold change threshold, we identified 599 DEGs, of which 225 were upregulated and 374 were downregulated in macroH2A1.1^−/−^ compared to macroH2A1.1^fl/fl^ controls (Additional file 2: Table S1). KEGG analysis revealed that these genes significantly over-represented the ribosome pathway, protein processing in the endoplasmic reticulum, and the cysteine/methionine metabolism pathways (Fig. 4a). The ribosome pathway was the most significantly enriched pathway in our experiment (Fig. 4b), with 16 differentially expressed ribosomal proteins with their functional location within the large (Rpl) and small (Rps) subunits. Furthermore, we found the broad functions of chromatin modification/remodeling, transcription, redox cell metabolism, cytoskeleton homeostasis, and cellular response to DNA damage stimulus among the top 20 most represented biological processes (Gene Ontology, GO) in macroH2A1.1^−/−^ HPCs (Fig. 4c). As defective ribosome biogenesis has been reported in MDS [30], we next asked whether macroH2A1.1-deficiency-driven changes in ribosomal protein gene expression underpin pathology in hematopoietic cells. We stably knocked down (KD) macroH2A1.1 mRNA in human promyelocytic leukemia HL-60 and monocytic THP-1 cell lines grown in suspension [31], using lentivirally transduced shRNAs against the *H2AFY* gene. Upon efficient specific silencing of the macroH2A1.1 transcript (without altering the levels of macroH2A1.2 isoform) (Additional file 1: Figure S3A, B), we measured transcript levels for a subset of Rpl (19, 29, 38) and Rps (15a, 21) genes, whose expression displayed > twofold change in our RNA-Seq analysis (Additional file 2: Table S1). MacroH2A1.1 KD led to decreased Rpl19 (fivefold), Rpl29 (twofold), Rpl38 (fivefold), Rps15a (sixfold), and Rps21 (twofold) mRNA levels in both HL-60 and THP-1 cells (Additional file 1: Figure S3C, D), consistent with our RNA-Seq findings made in macroH2A1.1^−/−^ mouse HPCs (Fig. 4). In order to obtain evidence that macroH2A1.1 deficiency-dependent decreased ribosomal protein gene expression may lead to defective ribosome biogenesis and protein synthesis, we assessed the steady-state 47S pre-rRNA level in macroH2A1.1^−/−^ HPCs, and in human HL-60 and THP-1 cells KD for macroH2A1.1. Under these conditions, a ~ 85% decrease in the relative amount of 47S pre-rRNA was observed in macroH2A1.1^−/−^ HPCs compared to macroH2A1.1^fl/fl^ HPCs (Fig. 5a). Similarly, a ~ 70% and ~ 60% decrease in the relative amount of 47S pre-rRNA was observed in HL-1 and in THP-1 cells, respectively (Fig. 5b). In addition, we performed a protein synthesis inhibition assay using puromycin, an antibiotic that competes by acting as an analog of the three-terminal end of aminoacyl-tRNA, disrupting protein synthesis. To this purpose, we performed a [3H]-leucine incorporation assay upon treatment with puromycin (0, 0.5, 2 μg/ml) for 72 h in HL-60 and THP-1 cells; our findings show that protein synthesis was significantly inhibited in HL-1 and THP1 cells even in the absence of puromycin, and it was further inhibited upon treatment with 0.5 μg/ml puromycin (Fig. 5c). Two micrograms per milliliter puromycin was lethal in both cell lines (Fig. 5c).

**Fig. 6:**
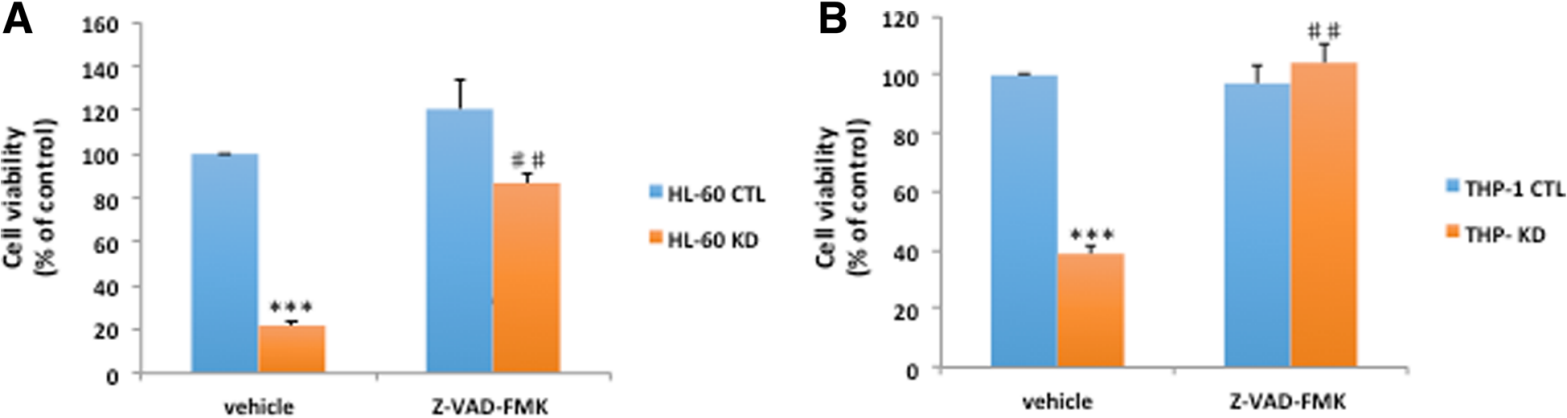
**a**, **b** MacroH2A1.1 knockdown (KD) affects cell viability in HL-60 and THP-1 cells. **a**, **b** Cell viability assay in HL-60 (**a**) and THP-1 (**b**) cells, incubated or not for 48 h in the presence of 10 μM pan caspase inhibitor Z-VAD-FMK. Data are presented as means relative to CTL cells, +/− SD, *n* = 4. ***p < 0.001 relative to CTL

**Fig. 7:**
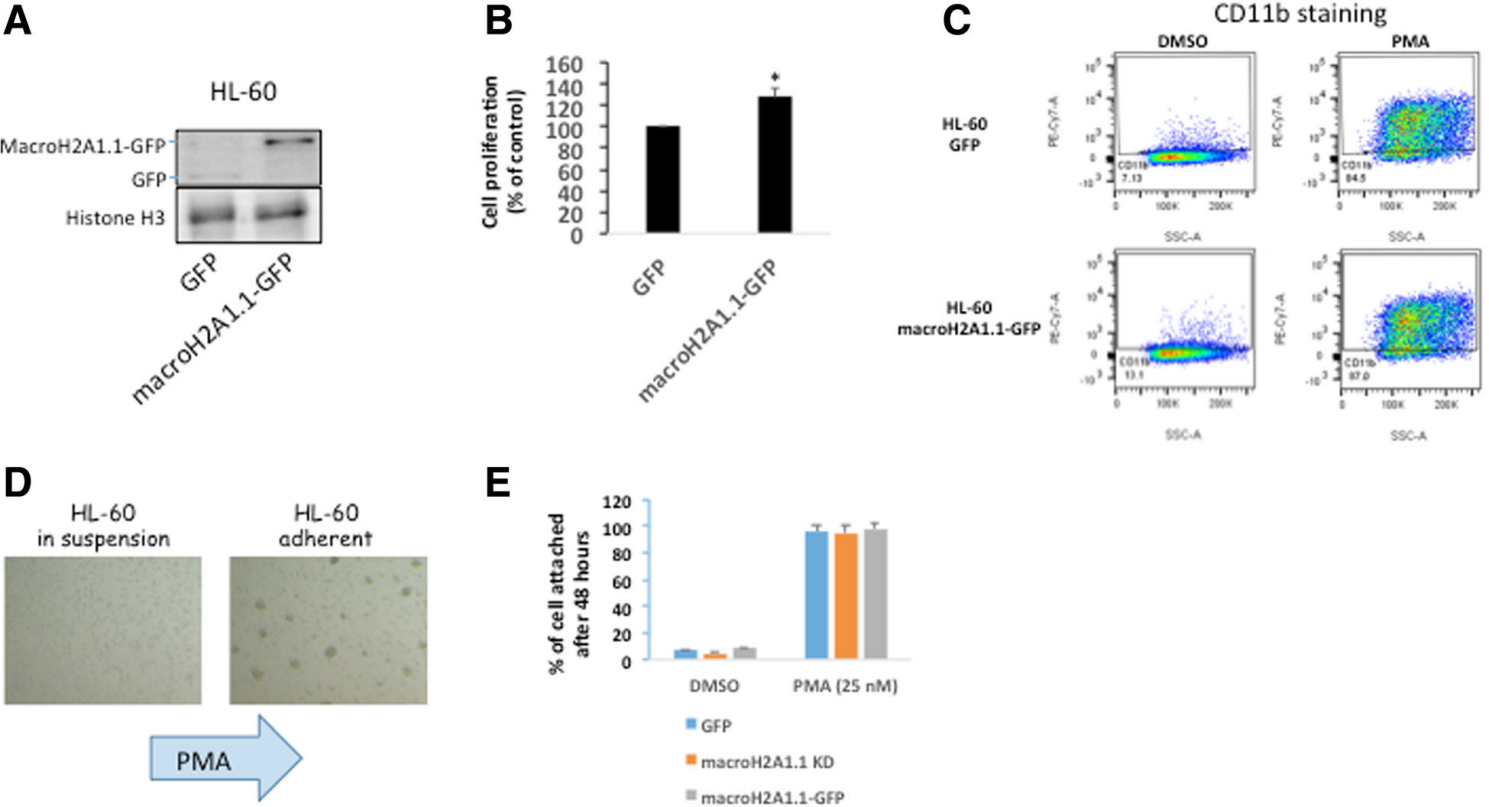
Effect of macroH2A1.1-GFP transgene overexpression, or macroH2A1.1 KD, on cell proliferation and differentiation. **a** Histones were extracted from HL-60 cells stably overexpressing a GFP vector or a stable macroH2A1-GFP transgene [9], and lysates were processed for immunoblotting with an anti-GFP antibody. Representative images are shown. **b** Cell proliferation assay in HL-60 cells, overexpressing GFP or macroH2A1.1-GFP, using labeling with carboxyfluorescein diacetate succinimidyl ester (CFSE) dye dilution by flow cytometry, 4 days upon adding the dye. Data are presented as the percentage of GFP cells, +/− SD, *n* = 4. **p < 0.01 relative to GFP. **c** FACS staining for pan-macrophage marker CD11b in HL-60 cells, overexpressing GFP or macroH2A1.1-GFP, upon treatment with Phorbol 12-myristate 13-acetate (PMA, 25 nM) or an equal amount of vehicle (DMSO) for 24 h. Representative gating is shown. **d** Representative images of HL-60 cells in suspension or adherent upon addition of PMA or DMSO as in **c**. **e** Adherent HL-60 cells, overexpressing GFP or macroH2A1.1-GFP, or KD for macroH2A1.1, upon treatment with PMA or DMSO as in **c**, were counted. Data are presented as the percentage of total cells (in suspension + adherent). Data are presented as means +/− SD, *n* = 4

In fact, macroH2A1.1 KD was associated with a ~ 60–80% decrease in cell viability of both HL-60 and THP-1 cells (Fig. 6a, b). This decrease was due to increased apoptosis, as a 48-h pre-incubation of the cells with the pan-caspase inhibitor Z-VAD-FMK (carbobenzoxy-valyl-alanyl-aspartyl-[O-methyl]-fluoromethylketone; 10 μM) completely rescued viability (Fig. 6a, b). Conversely, stable lentivirus-mediated overexpression of macroH2A1.1 fused to GFP(9) in HL-60 cells (Fig. 7a) led to increased cell proliferation (Fig. 7b). Treating HL-60 cells with phorbol 12-myristate 13-acetate (PMA) drives their differentiation toward a monocytic lineage [32]; however, here, we found that this process was apparently unaffected by macroH2A1.1 overexpression, with over-expressing cells successfully upregulating CD11b expression (Fig. 7c) and becoming adherent (Fig. 7d, e). PMA-driven differentiation was also unaffected by KD of macroH2A1.1 in the population of cells that remained viable (Fig. 7e).

### MacroH2A1 isoforms’ transcript levels are decreased in the BM of MDS patients carrying a 5q deletion or U2AF1 S34F mutation

**Fig. 8:**
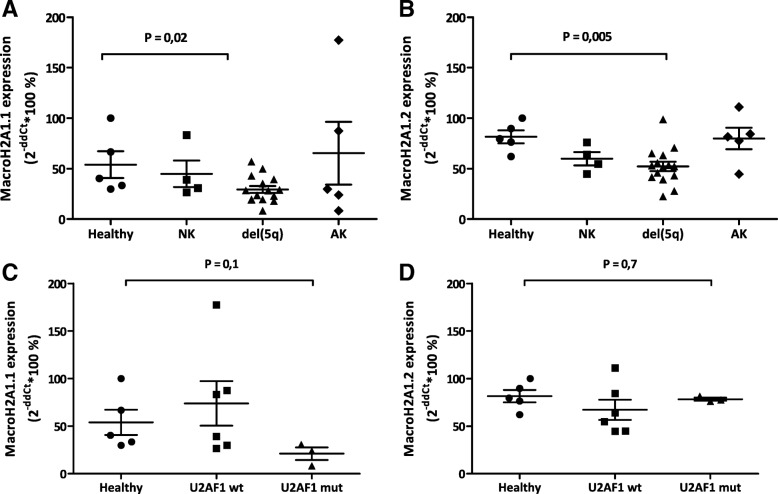
Expression analysis of two macroH2A1 transcript variants in MDS cells. **a**, **b** Relative mRNA expression of macroH2A1.1 (**a**) or macroH2A1.2 (**b**) in bone marrow samples from healthy individuals (*n* = 5), MDS patients with a 5q deletion (del5q; *n* = 15), or without del5q (NK, normal karyotype; *n* = 4), and MDS patients carrying other different chromosomal aberrations (AK, abnormal karyotype; *n* = 5). **c**, **d** MacroH2A1.1 and macroH2A1.2 mRNA levels were also compared between healthy individuals, MDS patients without the *U2AF1 S34F* mutation (wild type, wt), and MDS patients with the *U2AF1 S34F* mutation (mut). The *p* values are indicated, compared to healthy individuals

We first compared the BM expression levels of macroH2A1.1 or macroH2A1.2 between sub-groups of MDS patients with different genetic abnormalities and healthy controls (*n* = 5). MDS patients (*n* = 24 total) were categorized as either having a normal karyotype (NK; *n* = 4); a 5q deletion (del(5q); *n* = 15), of which seven also additional cytogenetic changes; or a non-del5q abnormal karyotype (AK; *n* = 5) (Additional file 3: Table S2). As expected, del(5q) patients expressed significantly lower macroH2A1.1 and macroH2A1.2 mRNA levels compared to healthy controls (*p* < 0.05), due to the loss of *H2AFY* located at 5q31.1 within the commonly-deleted 5q region (Fig. 8a, b). By contrast, NK and AK MDS patients showed normal macroH2A1.1/macroH2A1.2 mRNA levels (Fig. 8a, b). Aberrant *H2AFY* splicing causing specifically reduced macroH2A1.1, but not macroH2A1.2, expression has been associated with the *U2AF1 S34F* mutation in MDS [6]. However, only three MDS patients in this cohort carried this mutation, and they were not del(5q) carriers (Additional file 3: Table S2). When comparing these U2AF1 mutants versus healthy subjects, we detected a decreased, albeit not significant, macroH2A1.1 but not macroH2A1.2 mRNA expression (Fig. 8c, d), according to previous studies [6]. These data provide evidence that macroH2A1.1 mRNA tends to decrease in the BM of MDS patients carrying the *U2AF1 S34F* mutation and that both macroH2A1.1 isoforms’ transcript levels are decreased in the BM of MDS patients who are del(5q) carriers.

## Conclusions

Here, we show that the histone variant macroH2A1.1, which is downregulated in a subset of patients with MDS, profoundly affects the survival and differentiation of murine HSCs/HPCs in vivo and human leukemic cell lines in vitro. We provide evidence that macroH2A1.1 is required for normal ribosomal protein gene expression in HPCs. Without proper expression of this histone variant, abundant cell death occurred in the hematopoietic cells. Uncovering the relationship between macroH2A1.1 depletion and defective ribosomal biogenesis in HSCs/HPCs provides a critical link between this epigenetic regulator and the molecular pathologies typical of MDS.

Our findings are in line with a recent report from Yip et al. [6]. These authors analyzed the role of the mutation S34F in the splicing factor U2AF1, which is frequent in MDS: *U2AF1 S34F* altered mRNA splicing of many transcripts and among those *H2AFY* (coding for macroH2A1.1 and macroH2A1.2). Only 3 out of 29 patients in our cohort displayed *U2AF1 S34F* mutation; in those individuals, we observed a not statistically significant tendency toward a decrease in macroH2A1.1, but not macroH2A1.2 mRNA level: analyses on larger cohorts are required to corroborate these data [6].

*H2AFY* is physically localized on the long arm of chromosome 5, implying a connection with the most common karyotypic abnormality in MDS—del(5q). As expected, both macroH2A1.1 and macroH2A1.2 mRNA levels were found decreased in the BM of del(5q) MDS patients, compared to healthy subjects.

MacroH2A1.1 but not macroH2A1.2, which is deficient in poly-ADP-ribose binding, is required for PARP-1 activation [16, 28, 33, 34]. MacroH2A1 and PARP1 activities have been independently implicated in ribosome assembly [35–37]. Mutational [38] and transcriptomic [39] approaches have not fully clarified yet the role of del(5q) in MDS pathogenesis; however, CSNK1A1 [40] and RPS14 [41] have been identified as strong promising therapeutic targets. Hematopoietic differences observed in macroH2A1.1 haploinsufficient and macroH2A1.1-deleted mice have been similarly reported in a murine model displaying conditional inactivation of CSNK1A1 [40]. In this model, CSNK1A1 haploinsufficiency induced HSC expansion, whereas homozygous deletion caused HSC failure [40]. If CSNK1A1 and macroH2A1.1 functionally interact in regulating, hematopoiesis remains to be established. The phenotype of our newly generated macroH2A1.1 haploinsufficient mouse recapitulates several features of human MDS, including anemia, neutropenia, expansion of the LT-HSC pool, and a marked level of susceptibility to irradiation and defective myelopoiesis [3].

Various transcriptional and epigenetic mechanisms contribute to HSC maintenance and stepwise differentiation to produce distinct hematopoietic lineages [42, 43], and macroH2A1.1 has only recently emerged as a novel epigenetic regulator of hematopoiesis. Silencing macroH2A1.1 expression in human progenitor cells alters erythroid and granulomonocytic differentiation, and the reintroduction of macroH2A1.1 in U2AF1 mutant BM cells rescues cell death and their differentiation potential [6]. Consistently, we found that in lineage-committed HL-60 (neutrophils) and THP-1 (monocytes) human myelomonocytic leukemia cell lines, KD for macroH2A1.1 led to massive reduction in proliferation and death, and that macroH2A1.1 haploinsufficiency, and to a lesser extent, its full deficiency, tends to decrease the frequency of CMPs and MEPs, and to increase GMPs in mice. Expanded GMPs characterize high-risk MDS [44]. Compared to in vitro shRNA-mediated silencing or full in vivo KO, we believe that the features of our newly generated macroH2A1.1 haploinsufficient mouse might be more akin to the pathological changes observed in MDS patients. In fact, we also detected a myeloid bias in mice that lack the full dose of macroH2A1.1: the hematopoietic cell-autonomous enrichment of myeloid Mac-1+ cells parallels the myeloid differentiation bias that is commonly observed in other MDS murine models and in human MDS. MDS is characterized on one hand by dysplastic myeloid expansion, with myeloid cells that have reduced ability to differentiate, and on the other hand by neutropenia, thrombocytopenia, and anemia [45–47]: we propose macroH2A1.1^−/−^ mouse as a useful tool for further mechanistic studies on MDS.

By characterizing the phenotype of macroH2A1.1-insufficient mice, we report also another primary in vivo role for macroH2A1.1 in hematopoiesis: our RNA-Seq analysis of HPCs from macroH2A1.1^−/−^ mice identified a substantial depletion of many transcripts [small ribosomal proteins (Rps) and large ribosomal proteins (Rpl)] involved in ribosome assembly, and this was confirmed in HL-60 and THP-1 myeloid cell lines. Moreover, we found that macroH2A1.1^−/−^ HPCs and HL-60/THP-1 KD for macroH2A1.1 display decreased levels of 47S pre-rRNA, a long primary transcript that is the precursor of three of the four ribosomal RNAs: 18S, 5.8S, and 28S. Within ribosome structure, the 18S rRNA assembles with 33 Rps to form the 40S ribosomal subunit or small subunit, while the 5S, 5.8S, and 28S rRNAs associate with 47 Rpl to assemble the 60S or large subunit. 47S pre-rRNA and Rps/Rpl levels might thus be connected events leading to defective ribosome biogenesis in macroH2A1.1^−/−^ HPCs. Although the causal role for ribosomal biogenesis in HSC maintenance is not fully understood, impaired ribosome biogenesis-induced nuclear stress, for instance, due to hemizygosity for genes encoding ribosomal proteins, is associated with the development of clinical entities collectively known as “ribosomopathies” which include several bone marrow failure syndromes, including MDS [48]. We speculate that macroH2A1.1 depletion in the hematopoietic system might have two deleterious effects. First, it alters HSC homeostasis through defective ribosomal production [30]: adult HSCs display low levels of global protein synthesis relative to HPCs and genetic perturbations that alter the dynamics of protein synthesis impair HSC function [49]. Our data suggesting that macroH2A1.1-dependent impairment in ribosome biogenesis relates to impaired HSC differentiation are consistent with Signer et al., who demonstrated that reduced ribosome function in Rpl24 mice reduced protein synthesis of 30% in HSCs and impaired HSC function [50].

Second, given the extensive Rpl and Rps expression heterogeneity in the hematopoietic system [51], macroH2A1.1^−/−^ might disrupt the regulation of hematopoietic lineage-specific ribosomal proteins that might be involved in lineage differentiation. Predicting the phenotypes of perturbed Rpl and Rps expression patterns remains challenging and is an area that warrants future research.

In summary, our study shows that a loss of macroH2A1.1, which affects a subset of MDS patients, has a critical role in the defective hematopoiesis and perturbed ribosomal biogenesis that are central to MDS pathology. By combining clinical data with a discrete mutant mouse model and in vitro studies of human cells, we identify macroH2A1.1 as a key determinant of the cellular and molecular features of MDS. These data justify the exploration of macroH2A1.1 and associated proteins as therapeutic targets in hematological malignancies.

## Methods

### Clinical and laboratory data of MDS patients

Bone marrow aspirates, clinical information, and routine laboratory data were collected from 24 patients diagnosed with MDS according to the revised World Health Organization criteria [52] and prior to commencement of treatment, at the University Hospital Brno. Basic MDS patients and cytogenetic characteristics are shown in Additional file 3: Table S2. *U2AF1* mutation analyses were performed as described previously [6].

### Human bone marrow cell isolation and sample processing

Red blood cells were depleted using ACK lysing buffer (NH_4_Cl 150 mM, KHCO_3_, 10 mM, Na_2_EDTA 0.1 mM, pH 7.2). White blood cells were further processed for RNA isolation. Total RNA was isolated using TriReagent (MRC, USA) according to the manufacturer’s instructions and the quality of RNA was assayed with an Agilent 2100 Bioanalyser (RNA 6000 Nano Assay; Agilent Technologies, USA).

### Animal models

Mice lacking macroH2A1.1 were generated as follows: a 12 kb segment of the murine H2AFY gene (introns 5–8) was subcloned from a BAC by recombineering into p15A-HSV tk-DTA-amp. A lacZ-neo cassette [53], flanked by loxP and rox sites at the 5′ and 3′ ends, respectively, was inserted into the intron between exons 6a and 6b, also by recombineering [54]. Another rox site was inserted upstream of exon 6a and another loxP site inserted downstream of exon 6b so that Dre/rox recombination [55] would remove exon 6a and the lacZ-neo cassette, and Cre/loxP recombination would remove exon 6b and the lacZ-neo cassette; thus, Cre recombination will eliminate macroH2A1.1 expression. Southern blotting of genomic NheI-digested DNA from individual ES-cell-derived clones with a 3′ probe was used to identify homologous recombinants (Additional file 1: Figure S1). A 12.3-kb DNA fragment corresponds to the wild-type macroH2A1.1 locus; integration of the loxP-flanked neomycin cassette 3′ of exon 6b introduced an additional NheI site, thus increasing the size of the NheI DNA fragment to 16.2 kb in the targeted allele (Additional file 1: Figure S1). Cre-mediated recombination resulted in a 3.9-kb NheI DNA fragment recognized by the 3′ probe, which is diagnostic of the macroH2A1.1 allele. The targeting of the macroH2A1.1 allele was performed by electroporation of A9 ES cells, which were then injected into C57BL/6 eight cell-stage embryos. The targeted macroH2A1^fl/fl^ mice were crossed to deleter HPRT-Cre mice (129S1/Sv-Hprt^tm1(CAG-cre)Mnn^/J), purchased from Jackson Laboratories, USA, to remove the loxP-flanked neomycin cassette and generate macroH2A1.1^fl/−^ mice (heterozygous, HET), respectively. Mice heterozygous for the macroH2A1.1 allele were further crossed to deleter Cre mice to generate the macroH2A1.1^−/−^ (knockout, KO) mice, respectively. All mice used were obtained after eight generations of back crossing on a C57Bl/6 genetic background. Mice were bred and maintained at the EMBL Mouse Biology Unit, Monterotondo, or at Plaisant Srl (Rome, Italy), in accordance with current Italian legislation (article 9, 27 January 1992, number 116) under a license from the Italian Health Ministry. The congenic C57BL/6-Ly5.1 mice were purchased from Charles River Laboratory. C57BL/6-Ly5.1/2 recipients were generated by intercrossing C57BL/6-Ly5.1 and C57Bl/6-Ly5.2 mice (Harlan, Italy).

### Bone marrow transplantation assays

Radiation chimeras were generated as described [56]. For competitive transplantation assays, a 1:3 mixture of BM cells from the donor macroH2A1.1^fl/fl^, macroH2A1.1 HET, or macroH2A1.1 KO mice (CD45.2^+^) and helper WT (CD45.1^+^) mice, respectively, was injected into the tail vein of the recipient (CD45.1/2^+^) mice. For noncompetitive transplantation assays, 1 × 10^6^ BM cells from macroH2A1.1^fl/fl^, macroH2A1.1 HET, or macroH2A1.1 KO mice (CD45.2^+^) were injected into the recipient mice. The recipient mice were treated with Baytril (Enrofloxacin, Bayer) in drinking water (17 mg/ml) for the duration of the experiment.

### Statistical analyses

Data are shown as means ± standard error of the mean (SEM). Groups were compared with either Student’s *t* test or the non-parametric Mann-Whitney *U* test, as appropriate, using GraphPad Prism Software (version 5.00 for Windows, San Diego, CA, USA): significance was *p* ≤ 0.05. Survival analyses of mice employed the Kaplan-Meier estimator.

## Additional files

Additional file 1: Supplemental Material and Methods. **Figure S1.** Conditional KO of macroH2A1.1 in mice. A. Upper panel. Targeting construct containing the sequence encoding mouse macroH2A1 (H2AFY), a loxP-flanked neomycin (neo) cassette 3′ of exon 6b (included in macroH2A1.2), and a rox-flanked cassette 3’of exon 6a (included in macroH2A1.1). Lower panel. Target construct upon Cre-mediated excision. B. Southern Blot strategy to screen for positive clones: genomic NheI-digested DNA from individual ES-cell-derived clones with a 3′ probe was used to identify homologous recombinants. A 12.3-kb DNA fragment corresponds to the wild-type macroH2A1.1 locus; integration of the loxP-flanked neomycin cassette 3′ of exon 6b introduced an additional NheI site, thus increasing the size of the NheI DNA fragment to 16.2-kb in the targeted allele. **Figure S2.** Peripheral blood counts and biochemical parameters in macroH2A1.1 Fl/- and 1 KO mice. Blood samples were collected into heparinized containers from wild type (WT), macroH2A1.1 Fl/Fl, macroH2A1.1 Fl/- and KO mice via tail vein. Data are expressed as mean ± SEM. * *p* < 0.05; **p < 0.01; ***p < 0.001 as compared to macroH2A1.1 Fl/Fl. N=12-15 per each group. **Figure S3.** Effect of macroH2A1.1 knock/down (KD) on ribosomal protein gene expression in HL-60 and THP-1 cells. A, B. RNA was extracted from HL-60 (A) or THP-1 (B) cells stably overexpressing a vector carrying scrambled shRNA (CTL) or a vector carrying shRNA for macroH2A1.1 (KD), and processed for qPCR using specific primers against macroH2A1.1 or macroH2A1.2 transcripts. C, D. RNA was extracted from HL-60 (C) or THP-1 (D) cells stably overexpressing a lentiviral vector carrying scrambled shRNA (CTL) or a vector carrying shRNA for macroH2A1 (KD), and processed for qPCR using specific primers against Rpl19, Rpl29, Rpl38, Rps15a and Rps21 transcripts. Data are presented as means relative to CTL cells, +/- SD, *n* = 4. *** *P* < 0.001 relative to CTL. (DOCX 680 kb)

Additional file 2: **Table S1.** Basic MDS patient/sample characteristics. Patients are classified according to the WHO classification 2016 ^13^. Excel file provided separately. MDS-SLD - MDS with single lineage dysplasia. MDS-MLD - MDS with multi-lineage dysplasia. MDS-RS-MLD - MDS with ring sideroblasts and multi-lineage dysplasia. MDS-EB-1 - MDS with excess blasts-1. MDS-EB-2 - MDS with excess blasts-2. 5q- syndrome - MDS with isolated del(5q). (XLSX 56 kb)

Additional file 3: **Table S2.** List of 599 transcripts displaying >1.5 fold change in hematopoietic progenitor cells (HPC) isolated from bone marrow of macroH2A1.1 KO *versus* Fl/Fl mice. (PDF 295 kb)

## Data Availability

The datasets used and/or analyzed during the current study are available from the corresponding author on reasonable request.

## Abbreviations

BM: Bone marrow
CMP: Common myeloid progenitor
GMP: Cranulocyte-macrophage progenitor
HSC: Hematopoietic stem cells
KO: Knockout
MDS: Myelodysplastic syndrome
MEP: Erythroid progenitor
RNA-Seq: RNA-sequencing
