# Supplementary material for: Deficiency and haploinsufficiency of histone macroH2A1.1 in mice recapitulate hematopoietic defects of human myelodysplastic syndrome
Source: Clin Epigenetics. 2019 Aug 22;11:121. doi: 10.1186/s13148-019-0724-z (PMC6704528; doi:10.1186/s13148-019-0724-z)
Supplement: Supplementary file 1 — Supplemental Material and Methods. Figure S1. Conditional KO of macroH2A1.1 in mice. A. Upper panel. Targeting construct containing the sequence encoding mouse macroH2A1 (H2AFY), a loxP-flanked neomycin (neo) cassette 3′ of exon 6b (included in macroH2A1.2), and a rox-flanked cassette 3’of exon 6a (included in macroH2A1.1). Lower panel. Target construct upon Cre-mediated excision. B. Southern Blot strategy to screen for positive clones: genomic NheI-digested DNA from individual ES-cell-derived clones with a 3′ probe was used to identify homologous recombinants. A 12.3-kb DNA fragment corresponds to the wild-type macroH2A1.1 locus; integration of the loxP-flanked neomycin cassette 3′ of exon 6b introduced an additional NheI site, thus increasing the size of the NheI DNA fragment to 16.2-kb in the targeted allele. Figure S2. Peripheral blood counts and biochemical parameters in macroH2A1.1 Fl/- and 1 KO mice. Blood samples were collected into heparinized containers from wild type (WT), macroH2A1.1 Fl/Fl, macroH2A1.1 Fl/- and KO mice via tail vein. Data are expressed as mean ± SEM. * p < 0.05; **p < 0.01; ***p < 0.001 as compared to macroH2A1.1 Fl/Fl. N=12-15 per each group. Figure S3. Effect of macroH2A1.1 knock/down (KD) on ribosomal protein gene expression in HL-60 and THP-1 cells. A, B. RNA was extracted from HL-60 (A) or THP-1 (B) cells stably overexpressing a vector carrying scrambled shRNA (CTL) or a vector carrying shRNA for macroH2A1.1 (KD), and processed for qPCR using specific primers against macroH2A1.1 or macroH2A1.2 transcripts. C, D. RNA was extracted from HL-60 (C) or THP-1 (D) cells stably overexpressing a lentiviral vector carrying scrambled shRNA (CTL) or a vector carrying shRNA for macroH2A1 (KD), and processed for qPCR using specific primers against Rpl19, Rpl29, Rpl38, Rps15a and Rps21 transcripts. Data are presented as means relative to CTL cells, +/- SD, n = 4. *** P < 0.001 relative to CTL. (DOCX 680 kb) [file 13148_2019_724_MOESM1_ESM.docx]

**Supplemental Material and Methods**

*Southern blotting*

DNA was restriction-digested, separated on a 0.8% agarose gel and transferred to a Hybond-XL membrane (Amersham) in alkaline solution (0.4M NaOH and 1.5M NaCl). The blot was neutralized in 2× SSC solution, UV-crosslinked at 150 mJ and then prehybridized for 2h in prehybridization solution (0.5M Na2HPO4, 1mM EDTA, 5% SDS and 3% BSA). A DNA probe was labelled with the Random Primers DNA Labeling System (Thermo Fisher Scientific) according to the manufacturer’s manual. The membrane was hybridized overnight, washed in 40mM Na2HPO4, 1mM EDTA and 5% SDS and exposed on a phosphor screen (Fujifilm).

*Cell lines and assays*

Stable expression of macroH2A1.1 tagged with GFP in leukemic HL-60 and THP-1 cells was achieved by lentiviral transduction [^1^](#_ENREF_1)^,^[^2^](#_ENREF_2). For shRNAs targeting H2AFY isoform 1.1 cloning, oligonucleotide sequence was TCGACAGTGATGCTGTCGT [^3^](#_ENREF_3). HL-60 and THP-1 parental lines were purchased from ATCC and cultured in RPMI supplemented with 10% fetal bovine serum (FBS), with 1% penicillin/streptomycin. Cell proliferation was measured using the CellTrace™ CFSE Cell Proliferation Kit Protocol (ThermoFisher, Czech Republic). Cell viability was assessed with trypan blue staining using Countess II FL Automated Cell Counter (Life Technologies), according to manufacturer’s instructions. Differentiation of HL-60 and THP-1 cells into macrophages was achieved using phorbol 12-myristate-13-acetate (PMA, 25-50 nM), as previously described [^4^](#_ENREF_4).

*Immunoblotting*

MEFs were lysed in RIPA buffer (Merck, Germany), and protein fractions were immunoblotted as previously described [^1^](#_ENREF_1). Histone extraction procedure from HL-60 and THP-1 cells was previously described [^1^](#_ENREF_1). Primary antibody against GFP, macroH2A1.1, macroH2A1.2, γ-H2AX and histone H3, were obtained from Cell Signaling.

*Mouse embryonic fibroblasts (MEFs)*

Primary mouse embryonic fibroblasts (MEFs) were derived from wild type or macroH2A1.1 KO embryos as previously described [^5^](#_ENREF_5). For immunofluorescence microscopy experiments MEFs of early passages (1 to 3) were utilized.

*Histology and immunohistochemistry*

Excised livers were fixed in 4% paraformaldehyde solution, dehydrated in serial alcohol solutions, embedded in paraffin, cut into 5-μ m-thick sections stained with H&E and examined under a light microscope (Nikon, Tokyo, Japan) ^[6](#_ENREF_6)^. Phosphorylation of the histone protein H2AX (γ-H2AX) was used an indicator of double strand breaks (DSB) in immunohistochemistry. Primary antibody against γ-H2AX was from Abcam (Germany). Immunohistochemistry protocol for mice livers and semi-quantitative evaluation were previously described [^7^](#_ENREF_7)^,^[^8^](#_ENREF_8).

*Antibodies and flow cytometry*

Flow cytometric analysis and cell sorting were performed essentially as described elsewhere ^[9](#_ENREF_9)^. Mice were sacrificed by cervical dislocation and bone marrows (BM) were collected from hind-leg bones in PBS containing 5% FCS (Life Technologies). All antibodies were purchased from eBiosciences (Thermofisher) or Biolegend. For HSC staining, Following red blood cells lysis, BM cells were Fc-blocked (CD16/CD32, clone 2.4G2, eBioscience) and incubated with PerCP-Cy5.5 conjugated antibodies against the lineage markers cocktail [CD3 (145-2C11), B220 (RA3-6B2), Mac-1 (M1/70), Gr-1 (RB6-8C5) and TER119 (Ter-119)] and eFluor780 –conjugated c-kit (2D8), Pe-Cy-7-conjugated Sca-1 (D7), APC-conjugated CD150 (TC15-12F12.2), Alexa-700-conjugated CD48 (HM48-1). In BM transplantation experiments, the antibodies against PE- conjugated CD45.1 (A20) and FITC-conjugated CD45.2 (104) were also added. For myeloid progenitor staining, cells were not Fc-blocked; and cells were stained with modified lineage cocktail (CD3 (145-2C11), B220 (RA3-6B2), Gr-1 (RB6-8C5), TER119 (Ter-119)) supplemented with Sca-1 (D7) and IL7Ra (A7R34) antibodies to exclude HSCs and common lymphoid progenitors, respectively, and APC-conjugated c-kit (2D8), PE-conjugated CD16/32 (2.4G2) and FITC-conjugated CD34 (RAM34) antibodies. Peripheral blood cells from competitively transplanted mice were analyzed as in ^[10](#_ENREF_10)^, stained with PE-conjugated B220 (RA3-6B2), CD8 (55-6.7) and CD4 (L3T4); APC-conjugated Mac1 (M1/70) and B220 (RA3-6B2), PE-Cy7-conjugated CD45.1 (104) and FITC-conjugated CD45.2 (A20). Analysis and cell sorting were done using a three-laser standard configuration FACSAria II (BD Biosciences) and an Attune NxT Acoustic Focusing Cytometer (equipped with Red, Blue and Violet lasers, Invitrogen). Analysis of flow cytometry data was performed with FlowJo software (Tree Star Inc).

*Gene expression*

Concerning MDS, 500 ng of total RNA was transcribed to cDNA (SuperScriptII; Thermo Fisher Scientific, USA) and analysed in triplicates by quantitative PCR. Quantitative PCR was performed using LightCycler 480® SYBR Green (Roche, Switzerland) in a QuantStudio™ 6 Flex System Instrument (Thermo Fisher Scientific, USA) according to the manufacturer´s instructions. Relative expression changes were calculated as 2−ddCt × 100%. Total RNA was also isolated from cultured cells or from mice tissue upon mechanical homogenization, using TRIzol (Invitrogen) and quantified using NanoDrop 1000 spectrophotometer (Thermo Scientific). Samples with an RNA integrity number between 9 and 10 were used for cDNA preparation (100 ng to 1 mg per sample). Quantitative PCR was performed using SYBR Green (SIGMA) in a LightCycler® Instrument (Roche, Switzerland), as described ^[11](#_ENREF_11)^. GAPDH transcript was used as internal control for normalization in all experiments. Mouse primer sequences were as follows: GAPDH, Forward 5’-CATCACTGCCACCCAGAAGACTG-3’, Reverse 5’-ATGCCAGTGAGCTTCCCGTTCAG-3’; macroH2A1.1, Forward 5’- ACCCGACAAACACTGACTTC-3’, Reverse 5’- CAGCTCCAGCTACCTCCAAG -3’; macroH2A1.2, Forward 5’-CCAGAAGCTGAACCTTATTCA-3’, Reverse 5’-CCTAGGTCATCTTTAAGGTC-3’; mouse rDNA, Forward 5’–TGTCTTGCCCCGCGTGTAAG -3’, Reverse 5-CGCTTACAAGAAACAGCGCG-3’.

Human primer sequences were as follows: GAPDH, Forward 5’-GGATTTGGTCGTATTGGG-3’, Reverse 5’-GGAAGATGGTGATGGGATT-3’; macroH2A1.1, Forward 5’-GTTCACCCGACAAACACTGA-3’, Reverse 5’-AGCAGCTCCAGCTACTTCCA-3’; macroH2A1.2, Forward 5’-GGTCCTCAGTGTAGCCCAAG-3’, Reverse 5’-AATGTGTCCGTCGTGGATCT-3’; RPL19, Forward 5’- GCCACATGTATCACAGCCTG-3’, Reverse 5’- GCGTGCTTCCTTGGTCTTAG-3’; RPL29, Forward 5’- GACTTGCCTACATTGCCCAC -3’, Reverse 5’- CCTTCTGTCCTCATGTTGGC-3’; RPL38, Forward 5’- TTTCGTCCTTTTCCCCGGTT-3’, Reverse 5’- GGTATCTGCTGCATCGAACT-3’; RPS15a, Forward 5’- GGCTAAACAAGTGTGGGGTG-3’, Reverse TCCCTCCTGTGTGTTTTCGT -3’; RPS21, Forward 5’- TCCGCTAGCAATCGCATCAT -3’, Reverse 5’- CATCTGACTCACCCATCCTACG-3’; human rDNA, Forward 5’–CCTGCTGTTCTCTCGCGCGTCCGAG-3’, Reverse 5’- AACGCCTGACACGCACGGCACGGAG-3’.

*RNA-Seq and bioinformatic analyses*

For RNA-Seq, total RNA was extracted from progenitor cells isolated from wild type and macroH2A1.1 KO mice (n=3 per group) with TRIzol Reagent (Invitrogen, Czech Republic). Indexed libraries were prepared from 2 mg/ea purified RNA with the TruSeq Total Stranded RNA Sample Prep Kit (Illumina, Cambridge, UK) according to the manufacturer's instructions. Libraries were quantified using the Agilent 2100 Bioanalyzer (Agilent Technologies, Santa Clara, USA) and pooled so that each index-tagged sample was present in equimolar amounts; the final concentration of the pooled samples was 2 nmol/L. Pooled samples were then subjected to cluster generation and sequencing using an Illumina HiSeq 2500 System (Illumina, Cambridge, UK) in a 2 x 100 paired-end format at a final concentration of 8 pmol/L. Short reads were aligned against the GRCm38 genome assembly using STAR (ver. 2.5.1a). Piled up reads were counted with *htseq-count* [^12^](#_ENREF_12). Normalization of reads counts and their comparisons were performed using the *edgeR* R package. Genes were considered differentially expressed between groups if their expression values differed by more than 1.5-folds, significantly (p≤ 0.05). Pathway and Gene Ontology enrichment analyses were performed by using DAVID (<https://david.ncifcrf.gov/>). Protein-to-protein interaction network was built using STRING (<https://string-db.org/>). All computations were performed with R 3.4.1 (R Core Team 2017).

*Measuring protein synthesis inhibition by [3H]-leucine incorporation*

HL-60 or THP-1 were seeded at 2 x 10^5^ cells per well in RPMI supplemented with 10% FBS and the plates were incubated overnight at 37°C to allow cells. Old media was removed and replaced with 0, 0.5 or 2 μg/ml of puromycin dissolved in RPMI with 10% FBS for 72 h in a 37°C humidified incubator. In the last 2h of incubation, cells were pulsed with 2 μCi of [3H]-leucine without removing puromycin. After 2 h and centrifugation of the cells in suspension, the media containing [3H]-leucine was removed by aspiration and the cells were washed with HBSS and transferred to scintillation vials. Radioactivity was determined using QuantaSmartTM software on a Tri-Carb scintillation counter (Perkin-Elmer).

**Supplemental References**

1. Borghesan M, Fusilli, C, Rappa, F, Panebianco, C, Rizzo, G, Oben, JA, Mazzoccoli, G, Faulkes, C, Pata, I, Agodi, A, Rezaee, F, Minogue, S, Warren, A, Peterson, A, Sedivy, JM, Douet, J, Buschbeck, M, Cappello, F, Mazza, T, Pazienza, V, Vinciguerra, M. DNA Hypomethylation and Histone Variant macroH2A1 Synergistically Attenuate Chemotherapy-Induced Senescence to Promote Hepatocellular Carcinoma Progression. *Cancer Res*. 2016;76(3):594-606. .

2. Lo Re O, Fusilli C, Rappa F, et al. Induction of cancer cell stemness by depletion of macrohistone H2A1 in hepatocellular carcinoma. *Hepatology*. 2017.

3. Yip BH, Steeples V, Repapi E, et al. The U2AF1S34F mutation induces lineage-specific splicing alterations in myelodysplastic syndromes. *J Clin Invest*. 2017;127(9):3557.

4. Chou SF, Chen HL, Lu SC. Up-regulation of human deoxyribonuclease II gene expression during myelomonocytic differentiation of HL-60 and THP-1 cells. *Biochem Biophys Res Commun*. 2002;296(1):48-53.

5. Xu J. Preparation, culture, and immortalization of mouse embryonic fibroblasts. *Curr Protoc Mol Biol*. 2005;Chapter 28:Unit 28 21.

6. Rappa F, Greco A, Podrini C, et al. Immunopositivity for histone macroH2A1 isoforms marks steatosis-associated hepatocellular carcinoma. *PLoS One*. 2013;8(1):e54458.

7. Pazienza V, Panebianco C, Rappa F, et al. Histone macroH2A1.2 promotes metabolic health and leanness by inhibiting adipogenesis. *Epigenetics Chromatin*. 2016;9:45.

8. Pindjakova J, Sartini C, Lo Re O, et al. Gut Dysbiosis and Adaptive Immune Response in Diet-induced Obesity vs. Systemic Inflammation. *Front Microbiol*. 2017;8:1157.

9. Bereshchenko O, Mancini E, Luciani L, Gambardella A, Riccardi C, Nerlov C. Pontin is essential for murine hematopoietic stem cell survival. *Haematologica*. 2012;97(9):1291-1294.

10. Bereshchenko O, Mancini E, Moore S, et al. Hematopoietic stem cell expansion precedes the generation of committed myeloid leukemia-initiating cells in C/EBPalpha mutant AML. *Cancer Cell*. 2009;16(5):390-400.

11. Benegiamo G, Vinciguerra M, Mazzoccoli G, Piepoli A, Andriulli A, Pazienza V. DNA methyltransferases 1 and 3b expression in Huh-7 cells expressing HCV core protein of different genotypes. *Dig Dis Sci*. 2012;57(6):1598-1603.

12. Anders S, Pyl PT, Huber W. HTSeq--a Python framework to work with high-throughput sequencing data. *Bioinformatics*. 2015;31(2):166-169.

13. Strupp C, Nachtkamp K, Hildebrandt B, et al. New proposals of the WHO working group (2016) for the diagnosis of myelodysplastic syndromes (MDS): Characteristics of refined MDS types. *Leuk Res*. 2017;57:78-84.

**Supplemental Figure and Tables**

**
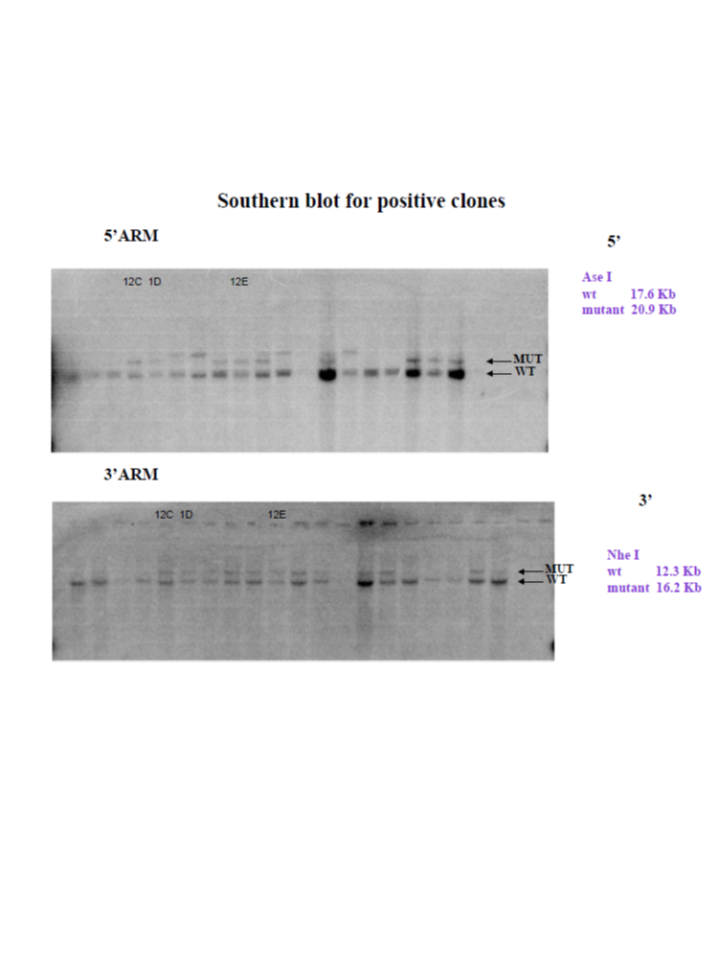
Supplemental Figure 1.** Conditional KO of macroH2A1.1 in mice. A. Upper panel. Targeting construct containing the sequence encoding mouse macroH2A1 (H2AFY), a loxP-flanked neomycin (neo) cassette 3′ of exon 6b (included in macroH2A1.2), and a rox-flanked cassette 3’of exon 6a (included in macroH2A1.1). Lower panel. Target construct upon Cre-mediated excision. B. Southern Blot strategy to screen for positive clones. Southern blotting of genomic NheI-digested DNA from individual ES-cell-derived clones with a 3′ probe was used to identify homologous recombinants. A 12.3-kb DNA fragment corresponds to the wild-type macroH2A1.1 locus; integration of the loxP-flanked neomycin cassette 3′ of exon 6b introduced an additional NheI site, thus increasing the size of the NheI DNA fragment to 16.2-kb in the targeted allele.

**
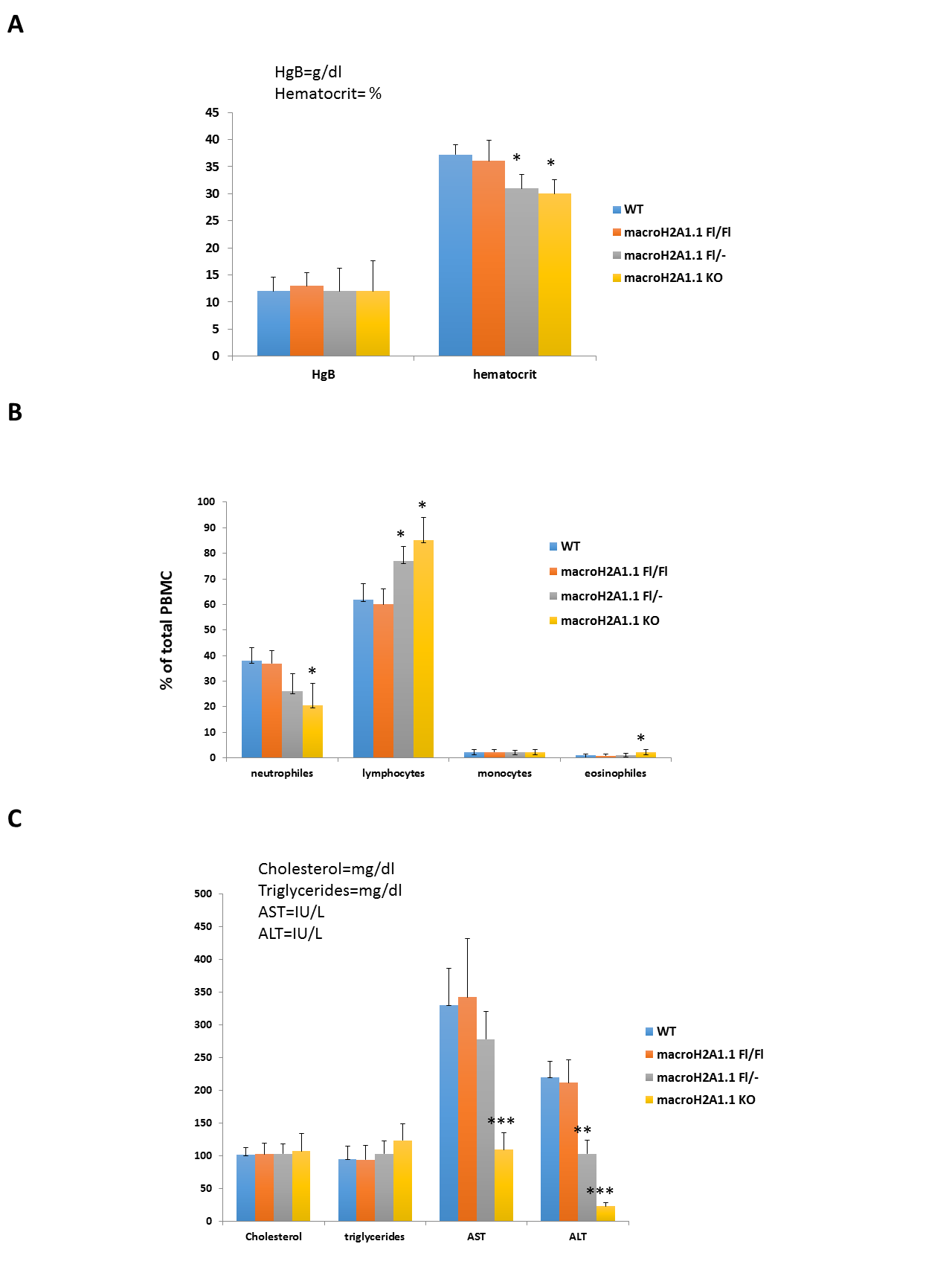
Supplemental Figure 2.** Peripheral blood counts and biochemical parameters in macroH2A1.1 Fl/- and macroH2A1.1 KO mice. Blood samples were collected into heparinized containers from wild type (WT), macroH2A1.1 Fl/Fl, macroH2A1.1 Fl/- and KO mice via tail vein. Data are expressed as mean ± SEM. * p < 0.05; **p<0.01; ***p<0.001 as compared to macroH2A1.1 Fl/Fl group. The number of mice per each group were 12-15.

**
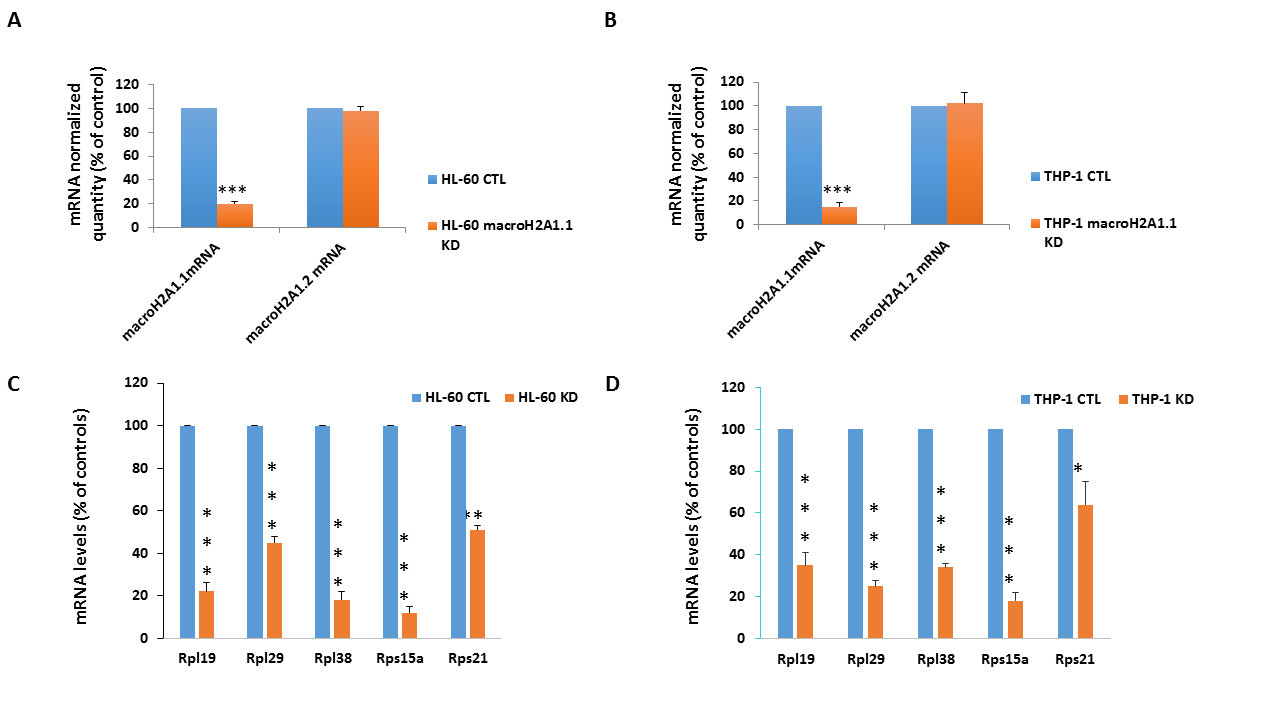
**

**Supplemental Figure 3.** Effect of macroH2A1.1 knock/down (KD) on ribosomal protein gene expression in HL-60 and THP-1 cells. A, B. RNA was extracted from HL-60 (A) or THP-1 (B) cells stably overexpressing a vector carrying scrambled shRNA (CTL) or a vector carrying shRNA for macroH2A1.1 (KD), and processed for qPCR using specific primers against macroH2A1.1 or macroH2A1.2 transcripts. C, D. RNA was extracted from HL-60 (C) or THP-1 (D) cells stably overexpressing a lentiviral vector carrying scrambled shRNA (CTL) or a vector carrying shRNA for macroH2A1 (KD), and processed for qPCR using specific primers against Rpl19, Rpl29, Rpl38, Rps15a and Rps21 transcripts. Data are presented as means relative to CTL cells, +/- SD, n=4. *** P<0.001 relative to CTL.

**Supplemental Table 1.** Basic MDS patient/sample characteristics. Patients are classified according to the WHO classification 2016 ^[13](#_ENREF_13)^. Excel file provided separately.

MDS-SLD - MDS with single lineage dysplasia

MDS-MLD - MDS with multi-lineage dysplasia

MDS-RS-MLD - MDS with ring sideroblasts and multi-lineage dysplasia

MDS-EB-1 - MDS with excess blasts-1

MDS-EB-2 - MDS with excess blasts-2

5q- syndrome - MDS with isolated del(5q)

**Supplemental Table 2.** List of 599 transcripts displaying >1.5 fold change in hematopoietic progenitor cells (HPC) isolated from bone marrow of macroH2A1.1 KO *versus* Fl/Fl mice.
