# Supplementary material for: Deficiency and haploinsufficiency of histone macroH2A1.1 in mice recapitulate hematopoietic defects of human myelodysplastic syndrome
Source: Clin Epigenetics. 2019 Aug 22;11:121. doi: 10.1186/s13148-019-0724-z (PMC6704528; doi:10.1186/s13148-019-0724-z)
Supplement: Supplementary file 3 — Table S2. List of 599 transcripts displaying >1.5 fold change in hematopoietic progenitor cells (HPC) isolated from bone marrow of macroH2A1.1 KO versus Fl/Fl mice. (PDF 295 kb) [file 13148_2019_724_MOESM3_ESM.pdf]

Supplemental Table 2

Basic MDS patient/sample characteristics															
Patient ID	Group	Sex	Age at diagnosis/ sampling - in case of Healthy Cont.	WHO classification	Therapy	Conventional cytogenetics	FISH				*TP53* Mutation status			*U2AF1* Mutation status	
							del(5q31)	del(7q31)	trisomy 8	other	*status*	*variant protein*	*variant protein*	*status*	*variant protein*
MDS058	healthy control	M	78		untreated	46,XY [9]	neg	neg	neg		wt			wt	
MDS064	healthy control	F	71		untreated	46,XX [11]	neg	neg	neg		wt			wt	
MDS120	healthy control	F	58		untreated	46,XX [15]	neg	neg	neg		wt			wt	
MDS313	healthy control	F	57		untreated	46,XX [14]	neg	neg	neg		wt			wt	
MDS318	healthy control	F	47		untreated	46,XY [16]	neg	neg	neg		wt			wt	
MDS009	MDS - normal karyotype	M	63	RCMD-RS	untreated	46,XY [9]	neg	neg	neg		mut	p.R181P		wt	
MDS028	MDS - normal karyotype	M	66	RCMD	untreated	46,XY [13]	neg	neg	neg		wt			wt	
MDS200	MDS - normal karyotype	M	71	RCMD	untreated	46,XY [17]	neg	neg	neg		wt			wt	
MDS208	MDS - normal karyotype	M	50	RCMD	untreated	46,XY [17]	neg	neg	neg		wt			mut	p.S34F
MDS029	MDS - deletion 5q	F	65	5q-	untreated	46,XX,del(5q) [8]/ 46,XX [8]	pos	neg	neg		wt			wt	
MDS030	MDS - deletion 5q	M	64	5q-	untreated	46,XY [18]/ 46,XY,del(5q) [1]	pos	neg	neg		wt			wt	
MDS031	MDS - deletion 5q	F	70	RA	untreated	46,XX [8]/ 73-89,complex changes including del(5q),der(7),del(20q) [8]	pos	pos	neg		mut	p.R273H		wt	
MDS035	MDS - deletion 5q	M	59	RAEB-2	untreated	46,X,-Y [2] / 46,XY [2]	pos	neg	neg	-Y	wt			wt	
MDS057	MDS - deletion 5q	F	62	RAEB-2	untreated	46,XX,del(5q) [10] / 46,XX [6]	pos	neg	neg		wt			wt	
MDS073	MDS - deletion 5q	F	70	5q-	untreated	46,XX,del(5q) [2]/ 46,XX [1]	pos	neg	neg		wt			wt	
MDS086	MDS - deletion 5q	F	63	5q-	untreated	46,XX,del(5q) [10]/ 46,XX [3]	pos	neg	neg		wt			wt	
MDS088	MDS - deletion 5q	M	72	RAEB-2	untreated	46,XY,del(5q) [5]/ 46,XY [12]	pos	pos	neg		mut	p.R213X		wt	
MDS094	MDS - deletion 5q	M	84	RAEB-1	untreated	40-46,XY,complex changes [11]/ 46,XY [9]	pos	neg	neg		mut	p.R175H		wt	
MDS127	MDS - deletion 5q	M	81	RAEB-1	untreated	41-48,XY,complex changes	pos	neg	neg		mut	p.G266R		wt	
MDS144	MDS - deletion 5q	M	54	5q-	untreated	46,XY,del(5q) [11]/ 46,XY [5]	pos	neg	neg		wt			wt	
MDS158	MDS - deletion 5q	F	70	RAEB-2	untreated	46,XX [11]	pos	neg	neg		wt			wt	
MDS190	MDS - deletion 5q	M	72	RAEB-2	untreated	41-49,XY,complex changes [14]/ 46,XY [5]	pos	pos	neg		mut	p.C141Y	p.R175G	wt	
MDS207	MDS - deletion 5q	M	76	RAEB-2	untreated	45-50,XY,complex changes [12]/ 46,XY [5]	pos	pos	neg	-7	mut	p.M246T		wt	
MDS323	MDS - deletion 5q	F	73	RAEB-2	untreated	46,XX,del(5)(q?13q33) [1]/ 42-46,XX,complex changes [8]/ 46,XX [10]	pos	neg	neg		mut	p.Y163C		wt	
MDS006	MDS - abnormal karyotype	M	66	RAEB-2	untreated	46,XY,t(2;3) [6]/ 46,XY [1]	neg	neg	neg	t(2;3)	wt			wt	
MDS053	MDS - abnormal karyotype	M	80	RCMD	untreated	46,XY [9]	neg	neg	neg	del(20q12)	wt			mut	p.S34F
MDS082	MDS - abnormal karyotype	M	58	RCMD	untreated	46,XY,der1 [10] / 46,XY [3]	neg	neg	neg		wt			mut	p.S34F
MDS182	MDS - abnormal karyotype	M	75	RCMD	untreated	46,XY,del(20)(q?12) [17]/ 46,XY /2/	neg	neg	neg	del(20q12)	wt			wt	
MDS302	MDS - abnormal karyotype	M	73	RAEB-2	untreated	44,XY,-7,del(13)(q?14),-18 [6]/ 46,XY [4]	neg	pos	neg	del(13q), -18	mut	p.C141Y		wt	
